# Galvanism in Paralysis of the Bladder

**Published:** 1849-06

**Authors:** 


*Galvanism in Paralysis of the Bladder.*—The manner of applying this remedy, adopted by M. Michon, of La Pitié Hospital, is very simple and is as follows: Two silver catheters or sounds are introduced, the one into the bladder, the other (the female) into the rectum, and being connected with the two poles of a simple electro-medical machine, the current is established and continued for a few minutes, when the sounds are withdrawn.

M. Michon, in two cases which he lately treated with success, used the remedy but once in the twenty-four hours.—*Ib.*

